# Multimodal Pain Management for Parsonage-Turner Syndrome in the Acute Rehabilitation Setting: A Case Report

**DOI:** 10.7759/cureus.43216

**Published:** 2023-08-09

**Authors:** Kashif Malik, Curren Giberson, Matthew Ballard, Nathan Camp

**Affiliations:** 1 Physical Medicine and Rehabilitation, Casa Colina Hospital and Centers for Healthcare, Pomona, USA

**Keywords:** multimodal analgesia, pts, parsonage turner syndrome, physical medicine and rehabilitation, turner, management, pain, neuropathic

## Abstract

Parsonage-Turner syndrome (PTS) is a rare neurological condition characterized by acute shoulder pain and novel neuromuscular weakness in the distribution of the brachial plexus. We present a case report highlighting the difficulties in the diagnosis and management of this condition. A multidisciplinary approach is often required to control this unique type of pain, consisting of neuropathic medications, non-steroidal anti-inflammatory drugs (NSAIDs) to control neuromuscular pain, and modalities provided by experienced physical therapists. Finally, a comprehensive, structured rehabilitation program focusing on the range of motion, neuromuscular re-education, and strengthening is imperative to regain function, reduce symptoms, and improve recovery.

## Introduction

Parsonage-Turner syndrome (PTS), also known as neuralgic amyotrophy or brachial neuritis, is a rare neurological condition that was coined in 1948. It is characterized by the onset of acute shoulder pain and new neuromuscular weakness. It is a rare condition, affecting 1-3 people per 100,000 [1], with a predilection towards males [2]. The syndrome is characterized by immune-mediated inflammation of the brachial plexus, causing pain and sensorimotor deficits in the affected extremity [3].

The exact pathophysiologic mechanism of PTS remains unclear. It is often triggered by an inciting event, such as a recent viral illness, immunization, or trauma [4]. The most common preceding factor was a recent viral illness, reported in 25% of patients prior to onset of PTS symptoms, while recent immunization was reported in 15% of patients prior to symptom onset. These statistics lend credibility to the hypothesis that immune-mediated inflammation is primarily responsible for this condition. However, a competing hypothesis is that ischemic changes lead to the classic presentation of PTS. This is supported by findings of perineural thickening, neovascularization, and focal fiber loss and the documented association between PTS and vasculitic conditions [5].

PTS classically presents with acute, severe shoulder pain that can radiate into the arm or back. The pain commonly resolves within one to two weeks and is followed by neuromuscular weakness that can involve any distribution of the brachial plexus [2]. However, the upper trunk and its distributaries, including the suprascapular, long thoracic, and axillary nerves, are most commonly affected [6,7]. The weakness may go unnoticed initially due to the patient’s unwillingness to move the affected limb during the acute painful phase. This leads to atrophy and loss of function of the hand, shoulder, and/or arm musculature, which can dramatically affect a patient’s activities of daily living. Sensory deficits can be seen but there is much variability in their prevalence in existing literature [8,9]. Fortunately, full recovery occurs in approximately 90% of patients [10].

The diagnosis of PTS can be challenging, as the differential diagnosis is broad. Symptoms may initially be attributed to conditions such as rotator cuff injury, frozen shoulder, or cervical disc disease, all of which can present with an acute painful shoulder. However, the history and physical examination, imaging, and electrodiagnostic studies can help to rule out other conditions and confirm a diagnosis of PTS. Given that PTS is believed to be an axonal process, the diagnosis is dependent on the electromyography (EMG) portion of the electrodiagnostic study. The most common finding is widespread denervation of muscles [9]. It is crucial to perform a thorough study of muscles in the upper extremity due to the variability in which PTS can present.

Pain management is a critical aspect of treating PTS, as the condition typically manifests with debilitating shoulder pain. The pain can vary in nature, but it is typically neuropathic with a musculoskeletal component. This pain can be difficult to control, and initial treatment often involves opiates, anti-inflammatory medications, and antiepileptic agents [7]. In this case report, we discuss the pain management needs of a patient affected with PTS in the setting of acute inpatient rehabilitation. 

## Case presentation

We present the case of a 53-year-old male with a history of traumatic brain injury (TBI) in June 2022 that was complicated by intracerebral hemorrhage and traumatic subdural hematoma. He initially presented to an outside hospital in December 2022 with shortness of breath and stridor and was given steroids. He was then transferred to a higher level of care for ear, nose, and throat (ENT) evaluation and ultimately required emergent intubation due to acute hypoxic respiratory failure. The patient had a prolonged hospital course due to methicillin-resistant *Staphylococcus aureus* (MRSA) and* Serratia* pneumonia and required IV antibiotics, including cefepime and vancomycin. His course was further complicated by his inability to wean from the ventilator, requiring tracheostomy and percutaneous endoscopy gastronomy tube placement, and bouts of agitation and delirium, including two instances where he decannulated himself. Lastly, he was diagnosed with a right middle lobe and subsegmental pulmonary emboli and placed on apixaban. He was then transferred to a skilled nursing facility (SNF) while he continued to recover.

He was eventually weaned to room air and determined to be an appropriate candidate for acute inpatient rehabilitation. Prior to his admission, it was reported that the patient could not move his left shoulder and lost strength in his left hand. This report was discussed with the patient after he arrived. He reported no deficits in his left arm prior to his transfer to the SNF. However, once he arrived at the skilled nursing facility, he recalled severe left shoulder pain and weakness in his left arm that was not associated with any new injury or trauma. Imaging from his initial admission was reviewed, including an MRI of his left brachial plexus that was negative for any acute pathology, an MRI brain that was unremarkable and negative for stroke, and an MRI of his cervical spine that did not show any acute process. 

His physical exam was notable for decreased sensation in a patchy distribution that included his left humerus overlying the deltoid muscle, the dorsum of his left hand, and all five fingers of his left hand. There was visible wasting of the left pectoralis major, supraspinatus, infraspinatus, and teres minor. On manual muscle testing, his strength was graded as a 3-/5 for left shoulder shrug, elbow flexion 3-/5, hand pronation 3-/5, hand to neutral 2/5, elbow extension 1/5, hand grip strength 2-/5, and wrist extension and flexion 2-/5. Deep tendon reflexes (DTRs) were assessed and graded 1 bilaterally for triceps, biceps, and brachioradialis. In comparison, there were no sensorimotor abnormalities detected on physical exam in his right upper extremity.

Throughout much of his admission, he experienced left upper extremity pain. He described it as a burning sensation that radiated from his left shoulder into his hand. The pain was constant and rated as 9 out of 10 on the conventional pain scale (CPS), although it was often worse at night. Upon arrival to acute rehab, we initiated gabapentin at a starting dose of 300 mg three times daily. On day two, the patient noted some improvement in his pain, demonstrated by a reduction in CPS score to 6 out of 10. However, the pain was still constant and, at times, a barrier to his rehabilitation. Next, duloxetine 30 mg daily was added as an adjunctive treatment and chosen because the patient had endorsed depressive symptoms since his initial injury. Ultimately, his gabapentin was increased to 600 mg three times daily, and after approximately 10 days, the patient reported his pain was a consistent 3 out of 10 and no longer interfering with his therapies. Lastly, topical diclofenac 2% gel was added to address any additional musculoskeletal pain he would experience from therapy. This regimen was effective for him and was well tolerated without any side effects.

**Figure 1:**
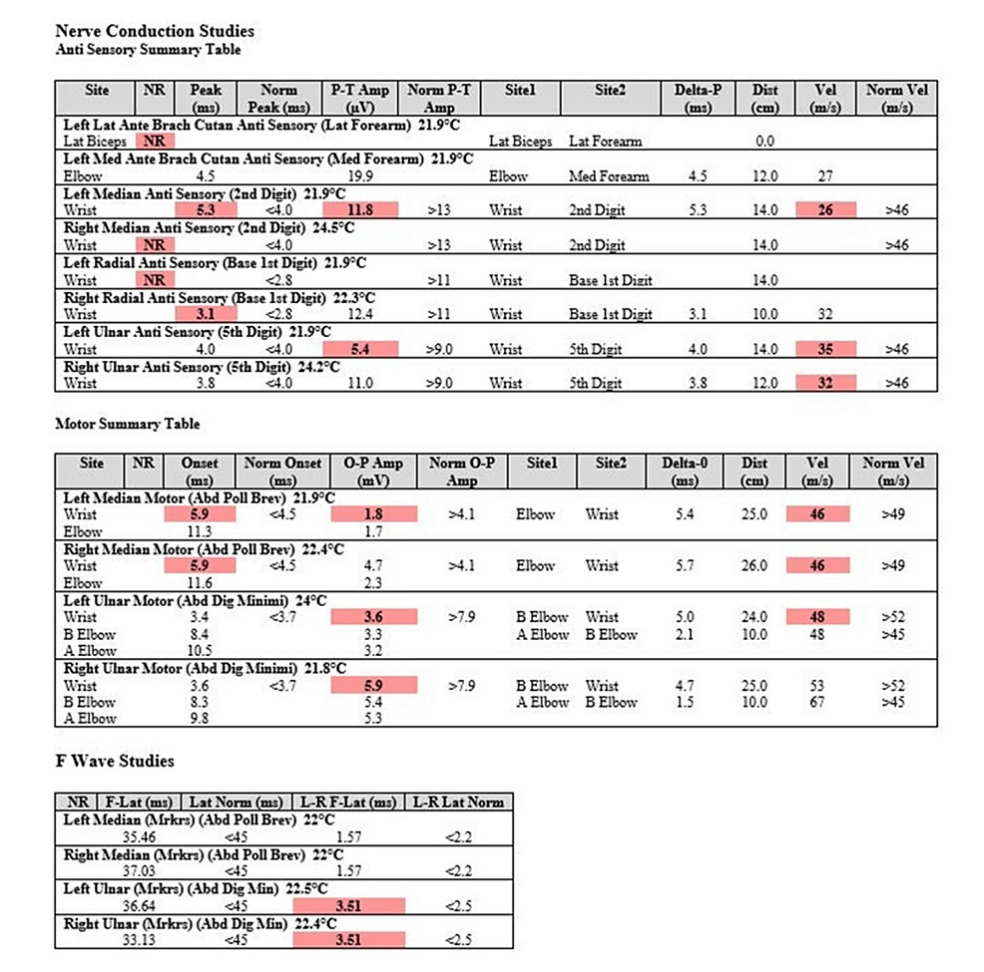
Results of a nerve conduction study in a patient with suspected Parsonage-Turner syndrome (PTS)

**Figure 2:**
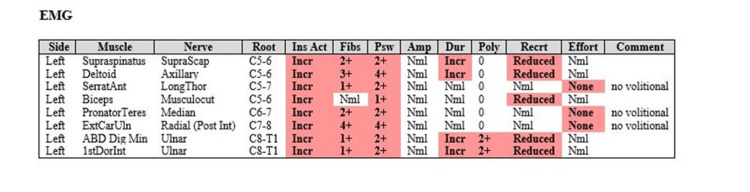
Results of an electromyogram in a patient with suspected Parsonage-Turner syndrome

Approximately three weeks after his initial symptoms, an electrodiagnostic study was performed. This included a nerve conduction test (Figure 1) and needle electromyogram (EMG) (Figure 2). Given the widespread involvement of different nerves in the left upper extremity, a single lesion cannot explain the abnormalities. Nerve conduction testing showed no response from the lateral antebrachial cutaneous sensory nerve, decreased velocity in the left median anti-sensory component, and no sensory from the right median nerve. The right and left medial motor studies both showed decreased velocity. Needle EMG showed that all muscles tested had increased insertional activity, fibrillation, and positive sharp waves, which is consistent with denervation and axonal demyelination. These findings are most consistent with a brachial plexopathy in the left upper extremity and median mononeuropathy in the right upper extremity. Additionally, lateral antebrachial cutaneous sensory nerve involvement is classic in PTS [11]. Since there was no clear explanation for his left upper extremity deficits and alternative diagnoses, such as cervical radiculopathy, mononeuropathy, and polyradiculopathy, were ruled out with the electrodiagnostic study, we concluded his deficits were due to PTS.

The patient went on to complete a comprehensive, multidisciplinary rehabilitation program with an emphasis on electrical stimulation to his left upper extremity. His therapy regimen consisted of exercises to maintain mobility via passive and active range of motion. Initially, the patient had very limited strength in his left upper extremity and was mobilized with passive and active-assist range of motion. As the patient progressed through his three week course, his strength improved significant and resistance training was employed to further his gains. Electrical stimulation was used aggressively to promote neuromuscular retraining from the beginning of the patient's admission. As the patient regained functional movement in his affected extremity, he began to work on activities of daily living such as toileting and progressed well.

Although pain and weakness were initial barriers to his therapy, he went on to make an excellent functional recovery, exemplified by the improvement in his functional independence measure (FIM) scores. At the time of admission, he was scored as setup/cleanup assist for eating and oral hygiene; partial/moderate assist for toileting hygiene and transferring, showering, and upper body dressing; substantial/maximum assist for lower body dressing; and dependent for putting on and removing footwear. After rehabilitation, he improved to an independent level in all the aforementioned categories. While he improved functional movement to the point of independence, he continued to have profound weakness in his left upper extremity and was instructed to follow up with outpatient treatment for continued therapies. A repeat electrodiagnostic study was scheduled three months in advance to better assess the recovery of his deficits.

## Discussion

Neuropathic pain is a common feature of PTS and can be difficult to manage with traditional pain medications. Most analgesic agents work by altering the way the nervous system processes pain signals through a descending pathway. Fortunately, our patient responded well to the combination of gabapentin and duloxetine to manage his neuropathic pain. However, many patients do not have such a favorable response and adequate pain control is an ongoing battle. This is why it is important to know what tools we have available in an inpatient setting to manage this unique type of pain. 

First and foremost of these tools are the neuropathic agents. These include, but are not limited to, anticonvulsants, tricyclic antidepressants (TCAs), and selective serotonin-norepinephrine reuptake inhibitors (SNRIs). The anticonvulsants gabapentin and pregabalin act on voltage-gated calcium channels to inhibit excitatory neurotransmitters such as glutamate. These are generally well tolerated and are often first-line treatments when it comes to neuropathic pain. If patients have side effects, such as sedation, or if their pain is not adequately controlled, other agents can be trialed. TCAs affect many different receptors in the brain including serotonin, norepinephrine, and N-methyl-D-aspartate (NMDA) receptors. Similarly, SNRIs inhibit the reuptake and increase the concentrations of serotonin and norepinephrine. While the mechanism is not completely understood, it is believed that these chemicals modulate the descending, inhibitory pain pathway at the raphe nucleus and dorsolateral funiculus [12]. These medications are first line and are often used in conjunction with traditional pain medications, such as opioids and NSAIDs, to manage patients with difficult-to-control neuropathic pain. 

When traditional medications are insufficient, alternatives must be considered. While our patient presented with predominantly neuropathic pain, there was a musculoskeletal component to it that often worsened after sessions of physical therapy. In this instance, topical diclofenac gel was applied to the shoulder girdle and scapular muscles with noted improvement in pain. Similarly, intra-articular pain and tendinopathy may contribute to the presentation and can be managed with corticosteroid injections, when appropriate. In severe cases, nerve blocks with a steroid or anesthetic can be performed to target the specific branch of the brachial plexus to provide short-term relief from unrelenting pain and aid in confirmation of the diagnosis [13]. Lastly, case studies have shown the efficacy of using prednisone [14] and immunotherapy [15] to reduce symptom burden. It is hypothesized that this reduces neuroinflammation, which may be a source of pain.

Due to the complex nature of PTS, a comprehensive approach must be taken towards pain management. Some of the most underutilized options are modalities provided by trained physical therapists. A physical therapy program for PTS is tailored to the unique deficits and symptoms experienced by each patient with the goal of restoring strength, mobility, and function. A program may include exercises focused on improving range of motion and posture, strengthening of the rotator cuff and other muscles of the shoulder girdle, and neuromuscular training exercises. Other modalities, such as iontophoresis and transcutaneous electrical nerve stimulation (TENS), can be applied during these sessions to manage localized pain. By working together with a team of experienced therapists, any physician managing a patient with pain due to PTS should have a multitude of tools in their belt.

## Conclusions

Overall, we present a case report of PTS and the acute pain management needs during the patient’s stay in inpatient rehab. PTS is a challenging diagnosis as it can be confused with many other upper extremity and CNS pathologies such as rotator cuff anomalies, adhesive capsulitis, peripheral nerve compression, cervical spondylosis, and cerebrovascular accidents. This condition causes profound pain and weakness that is debilitating to patients and requires a multimodal pain regimen combined with therapies to maximize recovery. Classic neuropathic medications such as gabapentin and duloxetine are effective in managing neuropathic pain, while NSAIDs and other medications are excellent in managing musculoskeletal pain that may result from therapies. Finally, a comprehensive, structured rehabilitation program focusing on the range of motion, neuromuscular re-education, and strengthening is imperative to regain function, reduce symptoms, and improve recovery.
